# Incidental Finding of Intraperitoneal Tuberculosis in a Patient Presenting with a Large Ovarian Mass

**DOI:** 10.1155/2018/9805702

**Published:** 2018-09-09

**Authors:** Ayla Green, Samer Elmasry, Himali Ihalagama, Saman Senaratne

**Affiliations:** Hervey Bay Hospital, Urraween Rd, Pialba, QLD 4655, Australia

## Abstract

A 52-year-old postmenopausal female of Filipino heritage presented with a three-day history of increasing abdominal bloating, vomiting, and fever. A CT scan revealed a 22 cm ovarian mass and ascites. Her laboratory results were unremarkable except for CA-125 and CA-19.9 which were slightly elevated. Due to suspicion of ovarian neoplasm, she underwent a laparotomy where multiple inflammatory deposits were observed throughout the pelvis. Histology confirmed florid granulomatous inflammation with caseous necrosis, and peritoneal fluid culture was positive for tuberculosis. She was treated with standard antimycobacterial therapy and made an uneventful recovery.

## 1. Introduction

Tuberculosis is a major global health issue, largely impacting the developing world, and yet it is a treatable disease. One-third of the world's population is infected with *Mycobacterium tuberculosis*, and 95% of cases are in the developing world [1]. Tuberculosis remains one of the top ten leading causes of death worldwide and has now surpassed human immunodeficiency virus as the leading cause of death secondary to an infectious disease [2]. Rates of tuberculosis in Australia have remained low, however high rates exist among indigenous people and those born overseas.

Intraperitoneal tuberculosis, or abdominopelvic tuberculosis (APTB), accounts for only 1-2% of cases of tuberculosis [3]. It most commonly arises from reactivation of latent tuberculosis via haematogenous spread from a primary lung infection [2]. The most common presenting features of APTB are abdominal pain, cachexia, fever, anorexia, abdominal distension, and ascites [3]. Without tissue sampling, it is difficult to distinguish from ovarian malignancy, especially in the presence of a raised CA-125 and ovarian mass.

## 2. Case Presentation

A 52-year-old postmenopausal female of Filipino origin presented to hospital with a three-day history of increasing abdominal bloating, vomiting, and fevers. She denied urinary or bowel symptoms. This patient had no significant past medical or family history and was a nonsmoker. She moved to Australia from the Philippines in 2015 and worked as a nurse in both countries.

On admission, she had a temperature of 39.9°C, a heart rate of 127, and a respiratory rate of 35. Her abdomen was markedly distended. There was a palpable tender mass in the right lower quadrant, with guarding and rebound tenderness.

**Figure 1:**
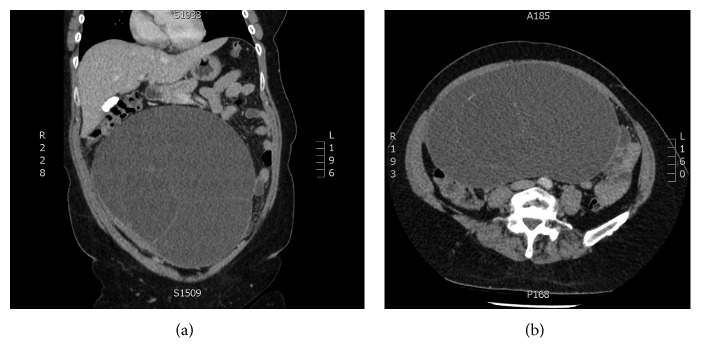
(a, b) CT abdomen showing large septated ovarian mass.

Initial investigations showed mildly deranged liver enzymes, an elevated CRP, and slightly elevated CA-125 and CA-19.9 (Table 1). CT scan showed a 22 × 13 cm multiseptated cystic lesion almost certainly of ovarian aetiology, as well as omental fat hazing, raising the possibility of an acute omental infarction (Figures 1(a) and 1(b)).

She was admitted for observation and intravenous antibiotics. Her fever resolved, and she was discharged home with a plan to follow-up in outpatient clinic for elective ovarian cystectomy.

**Figure 2:**
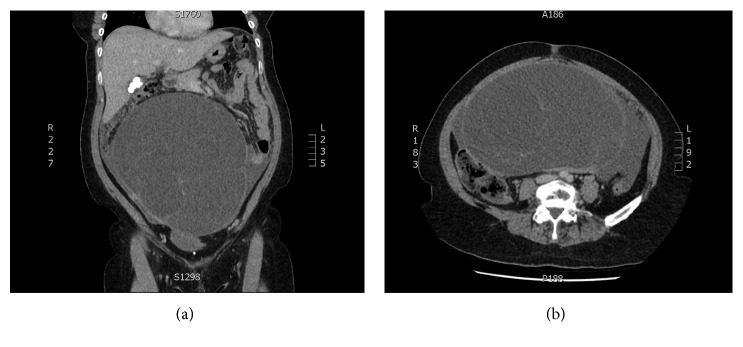
(a, b) Follow-up CT showing ruptured ovarian cyst.

Ten days later, the patient re-presented to hospital with severe abdominal pain and ongoing fevers. Repeat laboratory results showed worsening liver enzymes and a further rise of CRP (Table 1). A repeat CT scan showed the large ovarian cyst had likely ruptured with new generalised ascites and peritoneal enhancement, concerning for disseminated disease (Figures 2(a) and 2(b)).

**Figure 3:**
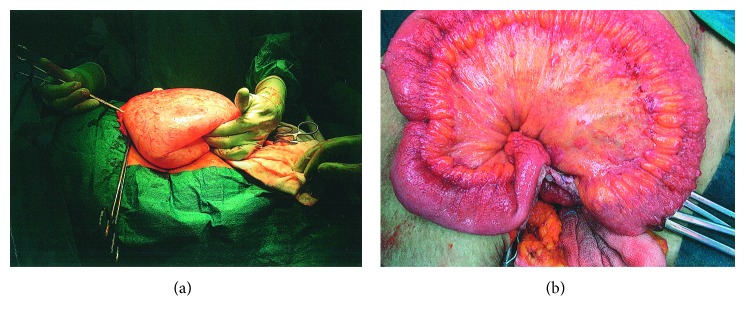
Intraoperative findings. (a) Large ovarian cyst with smooth surface; (b) characteristic nodules on surface of omentum and inflammatory changes of small bowel.

The patient underwent emergency laparotomy with total abdominal hysterectomy, bilateral salpingo-oophorectomy, infracolic omentectomy, and appendectomy. The cyst had a small leak, and 3.5 L of fluid was drained from the cyst and sent for histology (Figure 3(a)). A further 200 ml of ascitic fluid was collected. Inflammatory changes on the surface of the pelvis, and multiple inflammatory deposits overlying small bowel mesentery were noted (Figure 3(b)). Histology of the cyst showed a benign mucinous cystadenoma. The uterus, ovarian cyst, omentum, and appendix contained florid granulomatous inflammation and caseous necrosis. Peritoneal fluid cultured *Mycobacterium tuberculosis*. A chest CT confirmed lymph node calcification consistent with previous tuberculosis, with no evidence of active infection. Her postoperative recovery was uneventful, and she was commenced on isoniazid, rifampicin, ethambutol, and pyrazinamide under guidance of the Infectious Disease team. The patient remained well at a recent 3-month follow-up.

## 3. Discussion

Intraperitoneal tuberculosis can cause a raised CA-125 mimicking ovarian malignancy [3]. In one study, 5.7% of the 138 patients suspected of having ovarian cancer actually had tuberculosis [3]. Women with APTB tend to be younger than the typical age for malignancy, averaging between 20 and 40 years old [4]. Noninvasive testing for tuberculosis remains a challenge. The tuberculin skin test has a low sensitivity, especially in cases of APTB. Currently, laparoscopy and biopsy is the preferred method of differentiating between tuberculosis and ovarian cancer, thus avoiding hysterectomy [5]. This can be especially beneficial in patients wishing to preserve their fertility [5].

As with pulmonary tuberculosis, first line treatment for APTB is a combination of rifampicin, isoniazid, pyrazinamide, and ethambutol for six months. However, there are emerging cases of multidrug-resistant tuberculosis, with 2-3% of cases resistant to at least isoniazid and rifampicin in Australia and 600,000 new cases of drug resistance worldwide in 2016. Unfortunately, while the majority of cases of tuberculosis are treatable, there remains a significant gap in access to early detection and treatment [2].

Diagnosis of APTB can be challenging as the symptoms are nonspecific and may mimic gynaecological malignancy, bowel disease, or other infectious diseases. Interestingly, our patient had both a large ovarian cyst and APTB. This necessitated laparotomy due to suspected ovarian malignancy. This case emphasizes the importance of considering rare conditions in order to achieve the best possible patient outcome.

## Figures and Tables

**Table 1:** Laboratory results.

	Admission 1	Admission 2	Reference range
Haemoglobin	132 g/L	115 g/L	(115–160)
White cell count	7.8 × 10^9^/L	7.9 × 10^9^/L	(4.0–11.0)
Platelets	374 × 10^9^/L	**627** **×** **10**^**9**^**/L**	(140–400)
C-reactive protein	**69** **mg/L**	**226** **mg/L**	(<5.0)
Creatinine	62 *μ*mol/L	54 *μ*mol/L	(45–90)
Albumin	44 g/L	**30** **g/L**	(35–50)
Bilirubin (total)	**33** ***μ*mol/L**	12 *μ*mol/L	(<20)
Bilirubin (conjugated)	**6** ***μ*mol/L**	<4 *μ*mol/L	(<4)
Alkaline phosphatase	**111** **U/L**	**402** **U/L**	(30–110)
Gamma-GT	**83** **U/L**	**268** **U/L**	(<38)
Alanine transaminase	25 U/L	**37** **U/L**	(<34)
Aspartate transaminase	*∗*	**70** **U/L**	(<31)
CA-125	**38** **kU/L**	—	(<35)
CA-19.9	**50** **kU/L**	—	(<35)
Carcinoembryonic Ag	1.9 *μ*g/L	—	(<5.0)
HE4	43 pmol/L	—	(<70)
ROMA	17.8%	—	(<25.3)

## References

[1] Tinelli A., Malvasi A., Vergara D. (2008). Abdominopelvic tuberculosis in gynaecology: laparoscopical and new laboratory findings. *Australian and New Zealand Journal of Obstetrics and Gynaecology*.

[2] World Health Organization (2018). *Global Tuberculosis Report 2017*.

[3] Chen-Hsuan W., Chan-Chao C., Chih-Wen T., Hung-Yaw C., Ou Y.-C., Lin H. (2011). Disseminated peritoneal tuberculosis simulating advanced ovarian cancer: a retrospective study of 17 cases. *Taiwanese Journal of Obstetrics and Gynecology*.

[4] Devil L., Tandon R., Goel P., Huria A., Saha P. (2012). Pelvic tuberculosis mimicking advanced ovarian malignancy. *Tropical Doctor*.

[5] Martins-Filho A., Crispim P. C. A., Etchebehere R. M., de Oliveira C. D. C. H. B., Murta E. F. C., Nomelini R. S. (2017). Abdominopelvic tuberculosis with a frozen section analysis consistent with ovarian cancer. *Case Reports in Infectious Diseases*.

